# BAY 41-2272 activates host defence against local and disseminated *Candida albicans* infections

**DOI:** 10.1590/0074-02760140255

**Published:** 2015-02

**Authors:** Paulo Vítor Soeiro-Pereira, Angela Falcai, Christina Arslanian Kubo, Edson Antunes, Antonio Condino-Neto

**Affiliations:** 1 Curso de Medicina, Universidade Federal do Maranhão, Pinheiro, MA, Brasil; 2 Laboratório de Imunologia, Centro de Ensino Universitário do Maranhão, São Luís, MA, Brasil; 3 Departamento de Imunologia, Instituto de Ciências Biomédicas, Universidade de São Paulo, São Paulo, SP, Brasil; 4 Departamento de Farmacologia, Universidade Estadual de Campinas, Campinas, SP, Brasil

**Keywords:** BAY 41-2272, sGC agonist, innate immunity, *C. albicans*, *S. aureus*, microbicidal activity

## Abstract

In our previous study, we have found that 5-cyclopropyl-2-[1-(2-fluoro-benzyl)-1H-pyrazolo[3,4-b]pyridine-3-yl]-pyrimidin-4-ylamine (BAY 41-2272), a guanylate cyclase agonist, activates human monocytes and the THP-1 cell line to produce the superoxide anion, increasing in vitro microbicidal activity, suggesting that this drug can be used to modulate immune functioning in primary immunodeficiency patients. In the present work, we investigated the potential of the in vivo administration of BAY 41-2272 for the treatment of Candida albicans and Staphylococcus aureus infections introduced via intraperitoneal and subcutaneous inoculation. We found that intraperitoneal treatment with BAY 41-2272 markedly increased macrophage-dependent cell influx to the peritoneum in addition to macrophage functions, such as spreading, zymosan particle phagocytosis and nitric oxide and phorbol myristate acetate-stimulated hydrogen peroxide production. Treatment with BAY 41-2272 was highly effective in reducing the death rate due to intraperitoneal inoculation of C. albicans, but not S. aureus. However, we found that in vitro stimulation of peritoneal macrophages with BAY 41-2272 markedly increased microbicidal activities against both pathogens. Our results show that the prevention of death by the treatment of C. albicans-infected mice with BAY 41-2272 might occur primarily by the modulation of the host immune response through macrophage activation.

Primary immunodeficiencies (PIDs) are a current topic of research, but the true incidence and prevalence of this group of diseases remains unknown. These diseases are associated with high morbidity and mortality, sequelae, high economic and social costs, psychological stress and family breakdown inherent to chronic and severe diseases (Modell et al. 2011). In recent years, significant advancements in the understanding of the diagnosis and immunopathological mechanisms of PID have been achieved, but progress in PID therapy has been limited.

At a minimum, recurring infections, which represent the most common feature of PID, can be treated with low or moderate doses of appropriate antibiotics, which can help to prevent permanent damage to affected organs, thus promoting the long-term survival of patients while improving their quality of life (Fried & Bonilla 2009). When appropriate, immunoglobulin therapy is an accepted treatment for some PID cases (Hoernes et al. 2011). Advanced treatments, such as those using interleukins, pegademase bovine and gamma interferon, can help in some complex cases (Errante et al. 2008, Booth & Gaspar 2009, Roy-Ghanta & Orange 2010). Bone marrow (BM) transplantation and gene therapy may be appropriate therapies for specific types of PID (Aiuti & Roncarolo 2009, Chiesa & Veys 2012). However, even with these treatments, the development of alternative therapies is necessary that aim to boost the immune system.

In recent years, our group has focused on drugs with the potential to modulate, activate or retrieve immune cell function. Our previous results using 5-cyclopropyl-2-[1-(2-fluoro-benzyl)-1H-pyrazolo[3,4-b]pyridine-3-yl]-pyrimidin-4-ylamine (BAY 41-2272) which is an agonist of soluble guanylate cyclase (sGC), were promising in terms of mononuclear phagocyte activation. BAY 41-2272 enhances classical phagocyte responses, such as nicotinamide adenine dinucleotide phosphate (NADPH) oxidase system function and reactive oxygen species (ROS) release and also has been shown to increase phagocytosis, microbicidal activity and cytokine production in peripheral blood monocytes and a THP-1 cell line (de Oliveira-Júnior et al. 2007 , Soeiro-Pereira et al. 2012).

In this study, we found that intraperitoneal treatment with BAY 41-2272 after infection remarkably reduced the death rate in mice. Further, this activity of BAY 41-2272 was dependent on the pathogen type, showing effectiveness against *Candida albicans*, but not *Staphylococcus aureus*. We also found that BAY 41-2272 induced an inflammatory response in the abdomen of mice (i.e., the accumulation of macrophages), promoted ROS production and enhanced antibacterial and antifungal in vitro activities. Our results suggest that BAY 41-2272 prevents the death of infected mice through the activation of host innate immunity, confirming its potential as an anti-infective drug.

## MATERIALS AND METHODS

*Chemicals* - BAY 41-2272 was provided by Pharma Research Centre, Bayer AG (Germany). Instant-Prov was obtained from NewProv Ltd (Brazil). Phorbol-myristate-acetate (PMA), transcutol (di-ethylene glycol ethyl ether), Cremophor-EL, thioglycollate, concanavalin A (Con A), carrageenan, zymosan A (*Saccharomyces cerevisiae*), crystal violet, horseradish peroxidase (HRP) type II, phenol red, (2-methoxy-4-nitro-5-sulphophenyl)-2H-tetrazolium-5-carboxanilide (MTT), Triton X-100 and dimethyl sulfoxide (DMSO) were purchased from Sigma (USA).

*Animals* - C3H/HePas male mice, weighing 20-25 g, were used [Animal Facility, University of São Paulo (USP)]. The animals were assigned to plastic cages (5 mice per cage) and housed under a 12-h light-dark cycle with access to unlimited supplies of filtered water and chow for one day before the initiation of the experiments. The studies were approved by the Animal Ethical Committee of the Institute of Biomedical Sciences of the USP, Brazil, according to the Guide for the Care and Use of Laboratory Animals prepared by the Institute of Laboratory Animal Resources, National Research Council and published by the National Academy Press (revised 1996).

*Animal treatments* - For the in vivo experiments, BAY 41-2272 was diluted in a transcutol, Cremophor-EL and water solution (10/20/70 ratio, vol/vol/vol) to a final concentration of 1 mg/mL, as previously described (Bischoff et al. 2003). The animals were then weighed and the drug doses were adjusted to 0.3, 1.0, 3.0 and 10.0 mg/kg. For in vitro stimulation, BAY 41-2272 diluted in a 0.7% DMSO solution was used at concentrations of 1.0 and 3.0 µM, according to Bischoff et al. (2003). Treatment with BAY 41-2272 was administered intraperitoneally (IP) for 48 h. A negative control group was injected with a saline solution and a positive control group was treated with 4% thioglycolate (Sigma) or Con A (0.5 mg/kg) (Sigma). An additional control group was treated with a dilution solution only.

The ex vivo experiments were performed using resident macrophages or macrophages obtained from mice treated with BAY 41-2272. Treatments with penicillin G (5 kU/kg) and tetracycline (1 mg/kg) or itraconazole (20 mg/kg) were also administered in the infection assays. To evaluate hydrogen peroxide (H_2_O_2_) production, an additional in vitro treatment with PMA (30 nM) (Sigma) was performed. Other reagents, treatments and models are described in the following specific methodologies.

Peritoneal and lymph node (LN) cell harvesting and BM cell counting. Mice were sacrificed by carbon dioxide (CO_2_) asphyxia at 48 h after the above-described treatment. Peritoneal cells were aseptically collected by washing the peritoneal cavity with 5 mL of sterile ice-cold phosphate-buffered saline (PBS) devoid of calcium and magnesium ions. Resident macrophages were obtained from untreated mice. For total cell determination, nine volumes of peritoneal cells were added to one volume of 0.05% crystal violet dissolved in 30% acetic acid and the cells were counted using a bright-line haemocytometer (Sigma). Differential cell counts were determined by cytospin preparations stained with Instant-Prov (NewProv).

After peritoneal fluid collection, the LN , spleen and femur were obtained. The LN and spleen were processed in 1 or 5 mL of PBS, respectively. The femur was washed with 1 mL PBS to obtain BM cells. For the total cell determination, nine volumes of peritoneal cells were added to one volume of 0.05% crystal violet dissolved in 30% acetic acid and the cells were counted using a bright-line haemocytometer (Sigma).

*Footpad oedema induction* - Animals were anaesthetised and injected subcutaneously (SC) with carrageenan (300 μg/paw in saline) into the right paw. Differences in the sizes of the injected vs. un-injected paws were used as an indicator of inflammation (paw oedema) (Winter et al. 1962). The properties of BAY 41-2272 were assessed by injecting various doses of this drug (0.01-1.0 mg kg^-1)^ IP at 48 h before the administration of carrageenan. Control mice were injected with same volume of a solvent (0.5 mL olive oil). Con A (100 mg kg^-1)^ served as a positive control. Inflammation was assessed at 60-min intervals during a 4-h period.

*Spreading assay* - A spreading assay was performed according to Rabinovitch et al. (1977). Peritoneal cell suspensions containing 2 × 10^6^ cells were centrifuged and suspended in 1 mL of 5 mM glucose in PBS. Fifty microlitres of cell suspension were layered on glass coverslips and incubated for 1 h at 37ºC. The coverslips were gently rinsed in PBS and the glass-adherent cells were fixed in 2.5% glutaraldehyde and examined with a phase contrast microscope at a 400X magnification. Two hundred macrophages were counted and scored as round or spread. An index of macrophage spreading (SI) was then calculated as follows: SI = number of spreading macrophages × 100)/200, i.e., SI = % of spreading macrophages.

*Zymosan phagocytosis assay* - A phagocytosis assay was performed according to Pinello et al. (2006). Peritoneal cell suspensions containing 2 × 10^6^ cells were centrifuged and suspended in 1 mL of RPMI medium. The cells were dispensed over round glass coverslips (20 mm) in six-well flat-bottomed microtest plates (Costar, USA) and the cultures were incubated at 37ºC for 20 min. After incubation, culture supernatants were aspirated and the non-adherent cells were removed. Adherent monolayers were rinsed with PBS. Subsequently, 1 mL of RPMI-1640 medium (Sigma) containing 5% heat-inactivated foetal bovine serum was added to the cultures. The cultures were maintained at 37ºC for 1 h in the presence of 1 mg/L* S. cerevisiae *zymosan (Sigma). The cultures were then washed with cold PBS to remove non-internalised particles. The cells were then fixed with 0.5% glutaraldehyde (Sigma). An average of 200 macrophages were counted using phase contrast microscopy to determine the phagocytic percentage. The phagocytosis index (PI) was calculated as follows: PI = the number of macrophages with phagocytic activity × 100)/200 adherent cells counted, i.e., PI = % of macrophages with at least two phagocytised zymosan particles.

*H* *_2_* *O* *_2_* *release and nitric oxide (NO) production* - H_2_O_2_ release and NO production were determined in a single macrophage sample using a previously described method (Cruz et al. 2007). To evaluate H_2_O_2_ release, a HRP-dependent phenol red oxidation microassay was used (Pick & Mizel 1981 ). For this assay, 2.0 x 10^6^ peritoneal cells were suspended in 1 mL of freshly prepared phenol red solution [ice-cold PBS containing 5.5 mM dextrose, 0.56 mM phenol red (Sigma) and 8.5 U/mL HRP type II (Sigma)]. One hundred microlitres of the cell suspension were added to each well and incubated with or without PMA (30 nM) (Sigma) for 1 h at 37ºC in a 5% CO_2_ humid atmosphere. Plates were centrifuged once at 150 *g* for 3 min and the supernatants were transferred to another plate. The reaction was stopped with 10 μL sodium hydroxide. Absorbance was measured at 620 nm with a microplate reader (MR 5000; Dynatech Laboratories Inc). The conversion of absorbance to μM of H_2_O_2_ was performed by comparison with a standard curve obtained with known concentrations of H_2_O_2_ (5-40 μM) diluted in RMPI medium (Pick & Keisari 1980).

Thereafter, the plates containing the cells were washed three times with PBS and the remaining adherent macrophages were cultured in 100 μL of RPMI-1640 medium (supplemented with 10 mM HEPES, 11 mM sodium bicarbonate, 100 U/mL penicillin, 100 μg/mL streptomycin, 2 mM L-glutamine, 23 mM L-asparagine, 1 mM folic acid, 0.1 mM pyruvic acid and 5% foetal calf serum) for 48 h at 37ºC in a 5% CO_2 _humid atmosphere. After the incubation, 50 μL of supernatants were collected and incubated with an equal volume of Griess reagent (1% sulfanilamide/0.1% naphthalene diamine dihydrochloride/2.5% phosphoric acid) for 10 min at room temperature (RT) to quantify the accumulation of nitrite (Ding et al. 1988). Absorbance was determined at 550 nm. The conversion of absorbance to μM of NO was performed by comparison to a standard curve obtained with known concentrations (5-60 μM) of sodium nitrite diluted in RPMI medium.

*Resistance of mice to C. albicans and S. aureus infections* - To assess the resistance of the BAY 41-2272-treated animals to *C. albicans* (ATCC 90028) and *S. aureus* (ATCC 25923), two models of infection were used as follows: (i) the inoculation of pathogens in the peritoneal cavity followed by survival rate evaluation and (ii) the inoculation of pathogens SC into the footpad of the animals. For the first model, the animals were inoculated IP with 0.5 × 10^6^ *C. albicans* blastospores or 5 × 10^6^ colony-forming units of *S. aureus.* Forty-eight hours from inoculation to the establishment of infection, the animals were also treated daily IP with BAY 41-2272 (1 or 3 mg/kg) or itraconazole (20 mg/kg) or penicillin G (5 KU/kg) and tetracycline (1 mg/kg) for three days. The survival rate of the animals was evaluated for 20 days from the first day of inoculation. We attempted to perform survival experiments in mice with less than 12 days of infection, but the results were reliable only for those infected for 20 days. For the second model, the animals were inoculated SC with the same concentrations of pathogens into the footpad of the left paw and the right paw served as the control. At 48 h from inoculation to the establishment of infection, the animals were treated daily IP or intralesionally (inoculated paw) with BAY 41-2272 (1 or 3 mg/kg) or itraconazole (20 mg/kg) or penicillin G (5 KU/kg) and tetracycline (1 mg/eg) for three days. Paw thickness was then measured after seven days from inoculation to assess the development of the lesion or infection.

*Ex vivo and in vitro peritoneal macrophage microbicidal activity* - To assess microbicidal activity, an MTT oxidation microassay was used after the incubation of the cells with bacteria or fungi. For this assay, two protocols were used to treat the peritoneal macrophages as follows: (i) the resident peritoneal cells were stimulated outside of the untreated animals (in vitro) and (ii) the cells were harvested from the animals treated as described previously in this protocol (ex vivo). After preparation, 2.5 × 10^5 ^cells were suspended in 200 µL of RPMI-1640 (without supplements) and distributed in a 96-well plate. The pathogens were then added at a 10:1 (pathogens:macrophages) ratio for *S. aureus* and a 2:1 ratio for *C. albicans*. Co-cultures were incubated for 2 h at 37ºC with 5% CO_2_. After incubation, the plate was centrifuged and the supernatants were collected and stored at -80ºC for the subsequent cytokine dosage assay. The cell pellets were washed twice with PBS to remove non-phagocytosed pathogens. After the washings, Triton X-100 (1.5%) was added for 10 min at RT to lyse the macrophages and release the pathogens. The cells were then washed twice with PBS to remove the Triton X-100, 100 µL of MTT (0.5 mg/mL) was added and they were incubated for another 2 h at RT away from light. After this incubation, 100 µL of DMSO was added and another 30-min incubation was performed to release formazan precipitate into the supernatant. After incubation, the plates were centrifuged (300 *g* for 3 min) and the supernatants were transferred to a new plate. Absorbance was determined at λ = 570 nm with a microplate reader (Dynatech Laboratories Inc). The conversion from absorbance to percentage of cell death was achieved with the following equation: 1 - (OD of sample - OD of 90% killing)/(OD of 0% killing - OD of 90% killing) × 100. This calculation was performed based on the concentrations of pathogens representing 100-10% of the total number of pathogens incubated with the cells.

*Data analysis and statistical procedures* - Statistical analyses were carried out using Prism 5.0 software (GraphPad). The data are expressed as the mean ± standard error of the means for the animals per group and were compared by one-way, two-way or repeated measures analysis of variance, followed by Bonferroni's post-test for multiple comparisons (Erceg-Hurn & Mirosevich 2008). Survival curves were compared with the log-rank (Cox-Mantel) test (Klein & Moeschberger 2003).

## RESULTS

**Fig. 1A:**
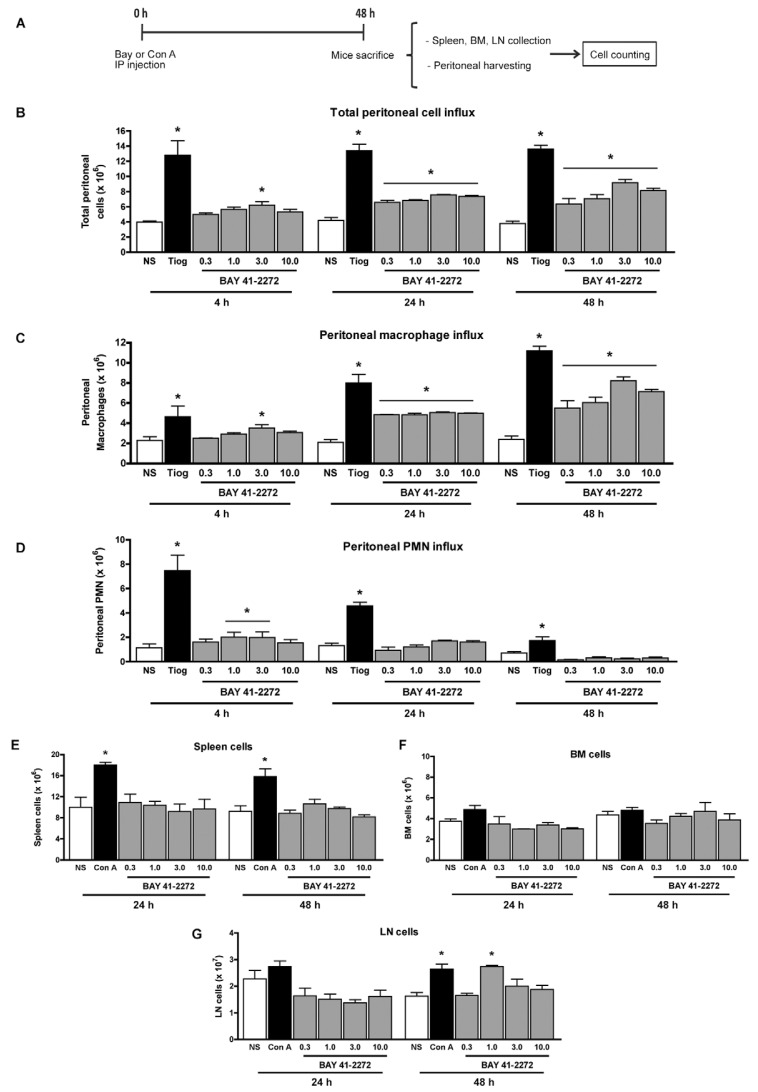
the diagram of this experimental protocol. 5-cyclopropyl-2-[1-(2-fluoro-benzyl)-1H-pyrazolo[3,4-b]pyridine-3-yl]-pyrimidin-4-ylamine (BAY 41-2272) increases cell influx into the peritoneal cavity cellularity, but not lymphoid organs and bone marrow (BM). C3H/HePas mice were treated intraperitoneally (IP) with thyoglycolate (4% in saline solution) or BAY 41-2272 (0.3, 1.0, 3.0, 10.0 mg/kg). At 48 h after treatment the animals were sacrificed to collect peritoneal cells for total (B) and differential [C: macrophages; D: polymorphonuclear (PMN)] cell counts and to collect the spleen (E), BM (F) and mesenteric lymph node (LN) (G). The organs were processed for determination of the total number of cells. The results are shown as the mean ± standard error of the means of number of cells from five experiments in triplicate. Asterisks mean p < 0.05 compared to non-stimulated group (NS). Con A: concanavalin A.

*Peritoneal cell influx and cell recruitment to lymphoid organs* - The mice were treated (or not) with BAY 41-2272 (0.3-10 mg/kg IP) for 48 h, after which the peritoneal cavity was harvested and the spleen, BM and LN were collected (Fig. 1A). The cellular distribution showed that treatment with BAY 41-2272 induced a significant increase in the total number of cells in the peritoneum compared with the control group (Fig. 1B). This cell population was composed primarily of macrophages (Fig. 1C), but the percentage of polymorphonuclear leukocytes (PMNs) was also elevated in the group treated with this drug (Fig. 1D). All vehicles used (transcutol, Cremophor-EL and water solution and DMSO) had no effect on this or the other assays performed in this study (data not shown).

There were no differences in the numbers of cells in the other lymphoid organs, such as the spleen, BM or mesenteric LN s, in the animals treated with BAY 41-2272 compared with the untreated animals (Fig. 1E-G). There was a trend of an increase in cell numbers only in the mesenteric LN s, indicating the recruitment of cells to this draining organ.

**Fig. 2A:**
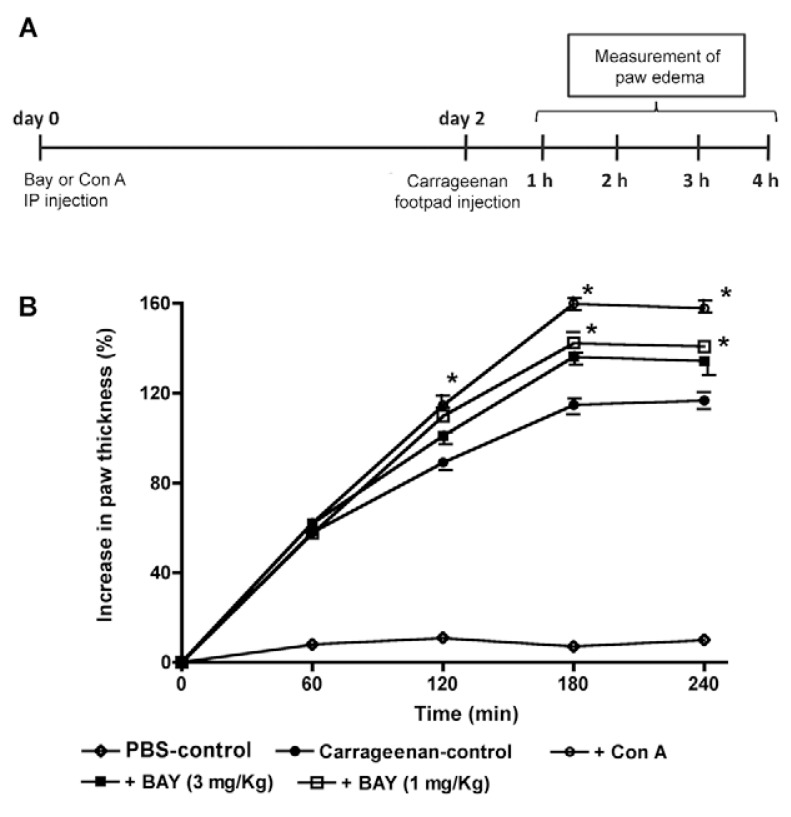
the diagram of this experimental protocol. 5-cyclopropyl-2-[1-(2-fluoro-benzyl)-1H-pyrazolo[3,4-b]pyridine-3-yl]-pyrimidin-4-ylamine (BAY 41-2272) potentiates inflammatory activity in a carrageenan model of paw oedema. C3H/HePas mice were treated intraperitoneally (IP) with concanavalin A (Con A) (0.5 mg/kg) or BAY 41-2272 (1.0 or 3.0 mg/kg). At 48 h after treatment, carrageenan (300 μg/paw) was injected in left footpad paw for measurement of oedema (paw size in mm) formed 3 h after the injection (B). The results are shown as the percentage of increase in paw size from the five experiments in triplicate. Asterisks mean p < 0.05 compared to carrageenan group (without pre-treatment with BAY 41-2272). PBS: phosphate-buffered saline.

*Carrageenan-induced footpad oedema* - To evaluate the effect of BAY 41-2272 on the inflammatory process we used a carrageenan-induced mouse paw oedema model. Mice were treated (or not) with BAY 41-2272 (0.3-10 mg/kg, IP) for 48 h, after which carrageenan (300 μg/paw) was injected into the footpad to measure oedema formation every hour for 4 h (Fig. 2A). Intraperitoneal pre-treatment with BAY 41-2272 significantly increased paw oedema, which was observed at 180 and 240 min after carrageenan injection (Fig. 2B). Similar data were observed for Con A. These results confirm the pro-inflammatory potential of BAY 41-2272.

**Fig. 3A:**
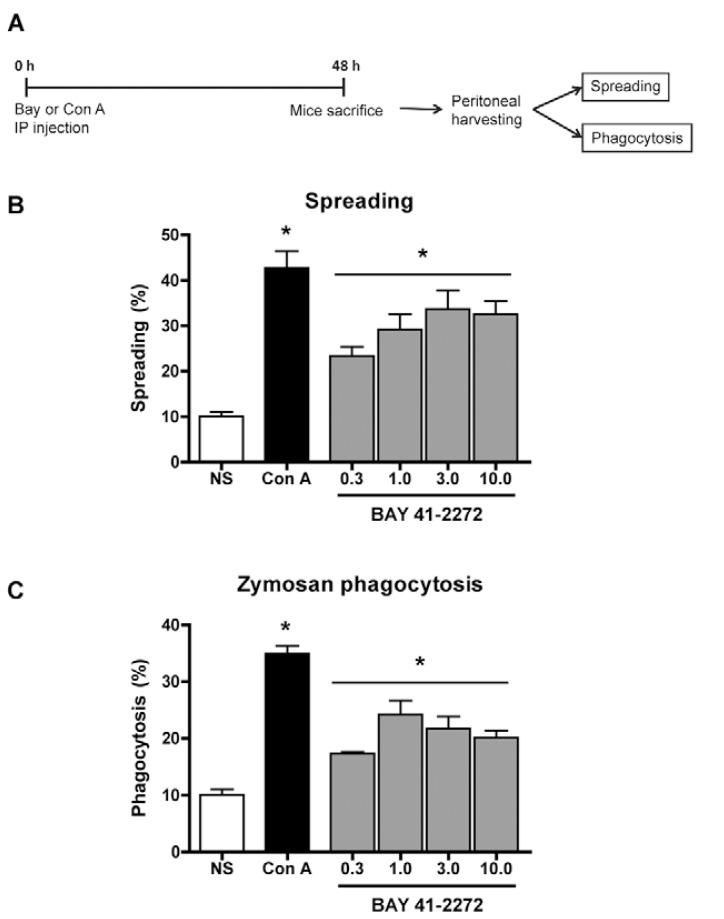
the diagram of this experimental protocol. 5-cyclopropyl-2-[1-(2-fluoro-benzyl)-1H-pyrazolo[3,4-b]pyridine-3-yl]-pyrimidin-4-ylamine (BAY 41-2272) increases ex vivo peritoneal macrophage spreading and phagocytic activity of zymosan. C3H/HePas mice were treated intraperitoneally (IP) with concanavalin A (Con A) (0.5 mg/kg) or BAY 41-2272 (0.1, 1.0, 3.0, 10.0 mg/kg). At 48 h after treatment, the animals were sacrificed and peritoneal cells (2 × 10^6^/mL) were harvested and incubated for 1 h at 37ºC on glass slides to define the percentage of spreading (B) or co-incubated with zymosan particles to set the percentage of phagocytosis (C) using phase contrast microscopy (400X). The results are shown as the mean ± standard error of the means of spreading or phagocytosis index from five experiments in triplicate. Asterisks mean p < 0.05 compared to non-stimulated group (NS).

*Ex vivo macrophage activation induced by BAY 41-2272 - spreading and phagocytosis* - With regard to the pro-inflammatory activity generated by BAY 41-2272, spreading and phagocytosis were assessed as markers of peritoneal macrophage activation. The peritoneal cavity of mice that were treated (or not) with BAY 41-2272 (0.3-10 mg/kg IP) for 48 h was harvested and peritoneal cells were incubated on glass slides to measure spreading or were incubated with zymosan to evaluate phagocytosis (Fig. 3A). The macrophages obtained from the BAY 41-2272-treated mice showed an increase in spreading compared with the untreated animals (Fig. 3B), which is consistent with their increased phagocytic activities (Fig. 3C).

**Fig. 4A:**
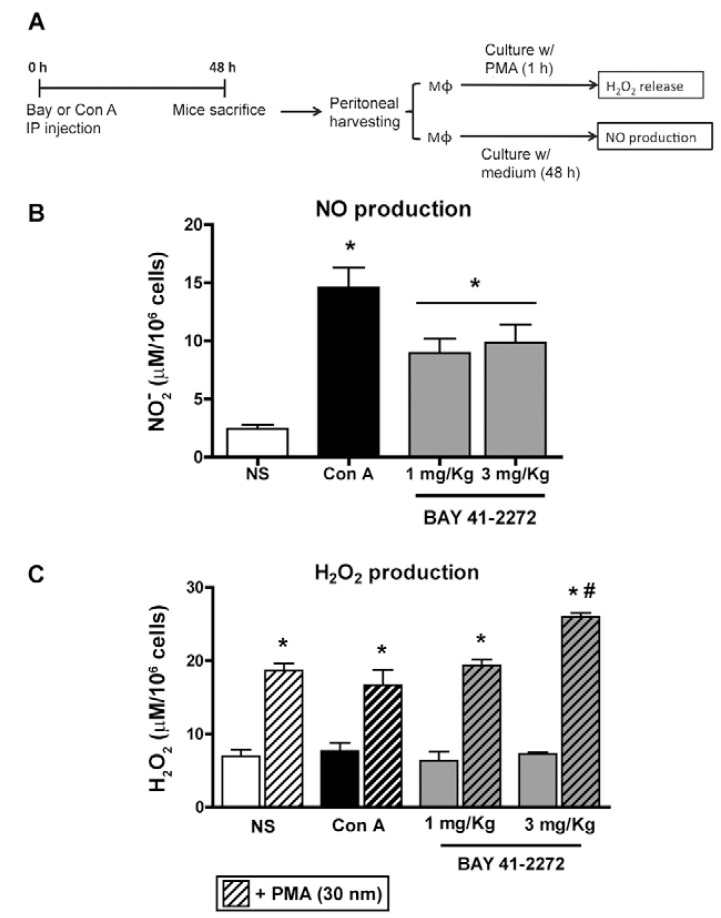
the diagram of this experimental protocol. 5-cyclopropyl-2-[1-(2-fluoro-benzyl)-1H-pyrazolo[3,4-b]pyridine-3-yl]-pyrimidin-4-ylamine (BAY 41-2272) increases ex vivo phorbol-myristate-acetate (PMA)-induced hydrogen peroxide (H_2_O_2_) release and nitric oxide (NO) production. C3H/HePas mice were treated intraperitoneally (IP) with concanavalin A (Con A) (0.5 mg/kg) or BAY 41-2272 (1.0, 3.0 mg/kg). At 48 h after treatment the animals were sacrificed and peritoneal cells (2 × 10^6^/mL) were harvested and treated or not with PMA (30 nM) for evaluation of H_2_O_2_ production (C) by phenol red oxidation assay. Cells obtained from the peritoneum (2 × 10^6^/mL) were also evaluated for NO production (B) by Griess reagent colorimetric assay. The results are shown as the mean ± standard error of the means of H_2_O_2 _or NO concentration from five experiments in triplicate. *: p < 0.05 compared to non-stimulated group (NS); #: p < 0.05 compared to NS treated with PMA.

*NO and H* *_2_* *O* *_2_* *production* - Mice were treated (or not) with BAY 41-2272 (0.3-10 mg/kg IP) for 48 h, after which the peritoneal cavity was harvested and peritoneal cells were incubated for 1 h with or without PMA (30 nM) to evaluate H_2_O_2_ release or were incubated for 48 h to evaluate NO production (Fig. 4A). It is known that phagocytosis and ROS release are related and are responsible for many antimicrobial responses. However, in this study, despite an increase in phagocytic activity, we did not observe alterations in spontaneous H_2_O_2_ release (Fig. 4C). However, the addition of PMA to the macrophage cultures from the BAY 41-2272-treated mice significantly increased the level of this metabolite (Fig. 4C).

Although pre-treatment did not induce the spontaneous release of H_2_O_2_, BAY 41-2272 significantly increased the spontaneous production of NO compared with the macrophages from the control group (Fig. 4B).

**Fig. 5A:**
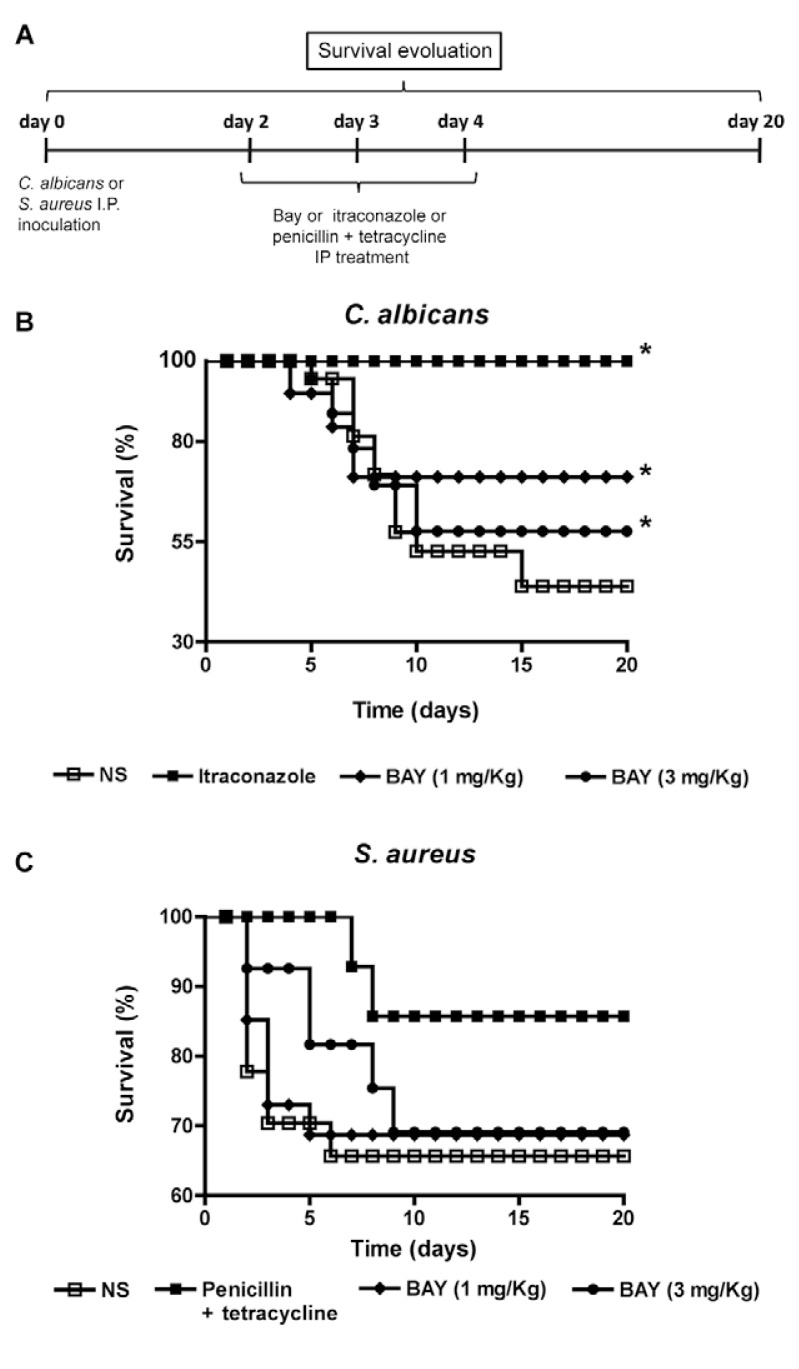
the diagram of this experimental protocol. Intraperitoneal treatment with 5-cyclopropyl-2-[1-(2-fluoro-benzyl)-1H-pyrazolo[3,4-b]pyridine-3-yl]-pyrimidin-4-lamine (BAY 41-2272) increases mice survival with *Candida albicans* infection (B), but not *Staphylococcus aureus* infection (C). C3H/HePas mice were inoculated intraperitoneally (IP), with *C. albicans* (0.5 × 10^6^ blastopores) or *S. aureus* (5 × 10^6^ colony-forming units). After 48 h, animals were treated or not with itraconazole (20 mg/kg) or penicillin G (5 kU/kg) and tetracycline (1 mg/kg) or BAY 41-2272 (1.0 or 3.0 mg/kg) for 72 h. The animals were followed for 20 days from the day of inoculation to assess survival rate. Results represent a survival curve of 20 animals per group and four experiments in triplicate. Asterisks mean p < 0.05 compared to non-stimulated group (NS).

*BAY 41-2272 increases survival of mice infected with fungi* - The increases in phagocytosis and microbicidal activity suggest that BAY 41-2272 has potential for the treatment for infections. Therefore, C3H/HePas mice were challenged with *C. albicans* and *S. aureus *and the survival rates of these animals were evaluated. Mice were inoculated with *C. albicans* or *S. aureus* IP and after 48 h, they were treated (or not) with either BAY 41-2272 (0.3-10 mg/kg IP) or itraconazole (20 mg/kg), penicillin G (5 KU/kg) and tetracycline (1 mg/kg) for three days. The survival rates of the animals were evaluated for 20 days (Fig. 5A).

The results showed that intraperitoneal treatment with BAY 41-2272 at 48 h after infection significantly increased the survival rate of the *C. albicans*-infected mice (Fig. 5B), but had no effects on that of the *S. aureus*-infected mice (Fig. 5C). In addition, as expected, itraconazole was completely effective in controlling *Candida* infection, maintaining the mouse survival rate at 100%.

**Fig. 6A:**
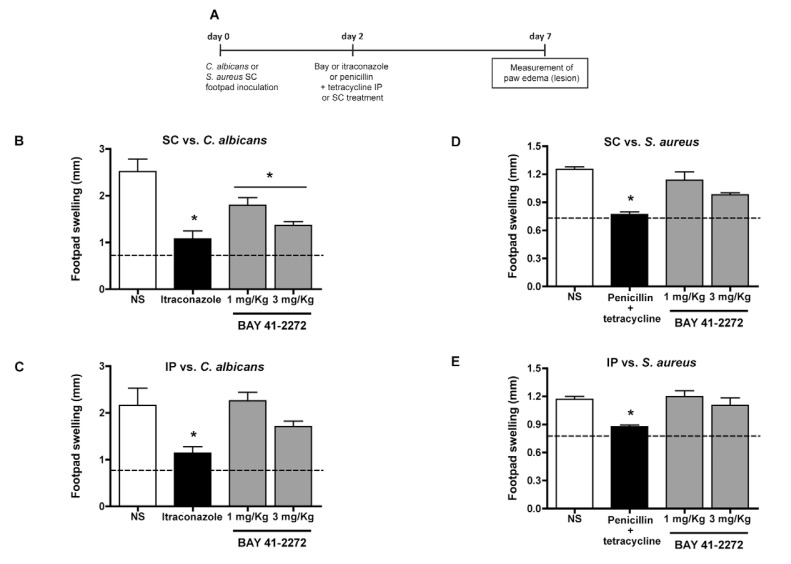
the diagram of this experimental protocol. Intraperitoneal and intralesional treatment with 5-cyclopropyl-2-[1-(2-fluoro-benzyl)-1H-pyrazolo[3,4-b]pyridine-3-yl]-pyrimidin-4-ylamine (BAY 41-2272) increases host protection against *Candida albicans*, but not by *Staphylococcus aureus* infection. C3H/HePas mice were subcutaneously inoculated in the footpad paw with *C. albicans* (0.5 × 10^6^ blastospores) (B, C) or *S. aureus* (5 x 10^6^ colony-forming units) (D, E). After 48 h, animals were untreated or treated (same paw infected) with itraconazole (20 mg/kg) or penicillin G (5 kU/kg) or BAY 41-2272 (1.0 or 3.0 mg/kg). Treatments were done intraperitoneally (IP) or subcutaneous (SC) (intralesional) routes. After seven days of the inoculum, paw thickness (mm) was measured. The results are shown as the mean ± standard error of the means of paw size from five experiments in triplicate. Asterisks mean p < 0.05 compared to respective infected, but not treated control. The dashed line represents the paws average thickness of animals treated with saline. NS: non-stimulated group.

*BAY 41-2272 increases mouse response against local C. albicans, but not S. aureus infection* - According to the observation that BAY 41-2272 increased the survival rate of *C. albicans-*infected mice*,* a model of infection in the animal footpad paw with the same pathogens was used (Fig. 6A). This protocol allowed for the evaluation of the direct effects of BAY 41-2272 on the site of infection (intralesional drug injection) and systemically (intraperitoneal drug administration).

Intralesional injection of BAY 41-2272 significantly reduced footpad swelling as induced by *C. albicans*, whereas the intraperitoneal treatment had no significant effect (Fig. 6B, C). The footpad swelling produced by *S. aureus* was not significantly altered by the subcutaneous or intraperitoneal BAY 41-2272 treatment (Fig. 6D, E).

**Fig. 7A:**
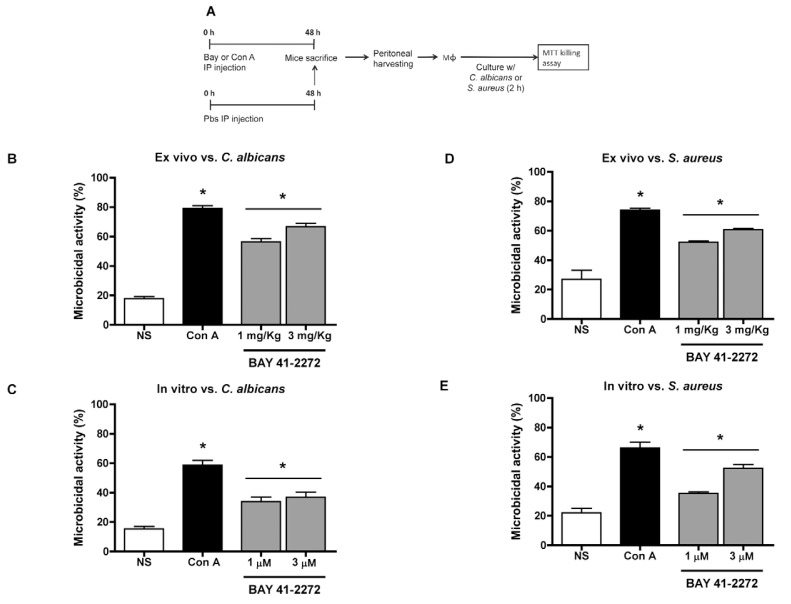
the diagram of this experimental protocol. In vitro and ex vivo treatment with 5-cyclopropyl-2-[1-(2-fluoro-benzyl)-1H-pyrazolo[3,4-b]pyridine-3-yl]-pyrimidin-4-ylamine (BAY 41-2272) increases peritoneal macrophages microbicidal activity. C3H/HePas resident macrophages were harvested from mice and treated in vitro with BAY 41-2272 (1 µM or 3 µM) or concanavalin A (Con A) (2 mg/mL). Macrophages from animals treated for 48 h *via* the intraperitoneal route (Con A, 0.5 mg/kg or BAY 41-2272 1.0 or 3.0 mg/kg) were also obtained. These cells (2 × 10^6)^ were incubated with *Candida albicans* (2:1) (B, D) or *Staphylococcus aureus* (10:1) (C, E) for 2 h. The microbicidal activity was assessed by (2-methoxy-4-nitro-5-sulphophenyl)-2H-tetrazolium-5-carboxanilide (MTT) reduction assay by bacteria recovered from macrophages. The results are shown as the mean ± standard error of the means of killing index from five experiments in triplicate. Asterisks mean p < 0.05 compared to respective infected, but not treated control. NS: non-stimulated group; PBS: phosphate-buffered saline.

*BAY 41-2272 increases in vitro and ex vivo microbicidal activities against C. albicans and S. aureus* - For the in vivo models of infection, BAY 41-2272 generated a better response to *C. albicans* than to *S. aureus*. Thus, the effect of the in vitro or ex vivo treatment of peritoneal macrophages with BAY 41-2272 was investigated by assessing its microbicidal activity in relation to both of these pathogens. Mice were treated (or not) with BAY 41-2272 (0.3-10 mg/kg IP) for 48 h, after which the peritoneal cavity was harvested and peritoneal cells were incubated with *C. albicans* or *S. aureus* for 2 h to assess microbicidal activity (Fig. 7A).

Our results confirmed the effectiveness of the BAY 41-2272 treatment, both in vitro and ex vivo, for increasing the microbicidal activity of peritoneal macrophages (Fig. 7). This treatment increased both fungal (Fig. 7B, C) and bacterial (Fig. 7D, E) killing in a similar way and almost as effectively as Con A (positive control). These results demonstrate the potential of BAY 41-2272 for treating fungal infections, specifically *C. albicans*, as shown by the in vivo model. Furthermore, these results showed that this treatment was effective in priming macrophages to respond against *S. aureus*.

## DISCUSSION

The present study demonstrated that in vivo treatment with BAY 41-2272 increased the host response against *C. albicans*. Moreover, BAY 41-2272 markedly increased macrophage functions, such as increased peritoneal influx, enhanced oxidative burst and NO release and increased zymosan phagocytosis and microbicidal activity against *C. albicans* and *S. aureus*. We also demonstrated that BAY 41-2272 treatment increased the survival of mice with *C. albicans* peritoneal infection. Our findings support the hypothesis that BAY 41-2272 activates the mononuclear phagocyte response and represents a potential application in the control of infections in immunocompromised hosts.

BAY 41-2272, especially after 48 h of administration, induced a significant increase in the total number of peritoneal cells, which primarily included macrophages. No significant increase in PMN number was observed, even during the first hours of treatment, indicating an atypical inflammatory reaction. There were no differences in cell number in other lymphoid organs, such as the spleen, BM and mesenteric LN s, in the animals treated with BAY 41-2272. These data suggest an on-site drug effect, primarily occurring through the activation of macrophages.

To clarify the extent of the activity of BAY 41-2272, we established a classical model of inflammation induced by carrageenan (Reiter et al. 1985). Our data revealed a pro-oedematous activity of BAY 41-2272 in a nonspecific inflammatory process. This pro-inflammatory effect is possibly enhanced by the involvement of 3,5-cyclic guanosine monophosphate (cGMP) in calcium flux (the control of actin and myosin fibres) and smooth muscle relaxation, leading to vasodilation and increased oedema (Schmidt et al. 2008, Morbidelli et al. 2010). It is important to note that carrageenan-induced paw oedema is largely dependent on PMN influx to the inflammatory site. Because the number of PMNs was unaltered by the BAY 41-2272 treatment, the increase in NO production can explain the carrageenan-induced oedema and NO has been previously described as a determinant of this response (Salvemini et al. 1996).

Along with evidence of inflammation generated by BAY 41-2272, we focused on the possibility of in vivo treatment with this compound to increase the immune response against pathogens. Therefore, we evaluated the function of peritoneal macrophages, which showed a significant increase in the potential for spreading and phagocytic activity. This increase in spreading is important for migration to inflammatory sites and also for phagocytosis (Tatefuji et al. 1996). Signal pathways activated by BAY 41-2272 are involved in cGMP generation and are associated with morphological changes required for migration, phagocytosis and spreading (VanUffelen et al. 1998, Borán & García 2007). The increase in BAY 41-2272-induced phagocytosis can be explained by protein kinase G activation, as has been recently shown by Bóran et al. (2008), promoting the rapid release and expansion of filaments in microglia cells and an increase in phagocytosis following treatment with ANP (particulate GC stimulator) and dibutyryl-cGMP (permeable analogue of cGMP).

It is known that phagocytosis and ROS release occur in almost all antimicrobial responses (Flannagan et al. 2009). However, despite the increase in phagocytic activity, we did not observe an increase in spontaneous H_2_O_2_ release. This result may be related to anti-oxidant mechanisms activated by cGMP and especially by cAMP, such as the activation of peroxisome proliferator-activated receptor-γ coactivator and glutathione peroxidase 1 (Lu et al. 2010 ).

However, this strong effect on the NADPH oxidase system appears to be temporary because the addition of PMA to macrophage cultures from treated mice induced the production of high levels of H_2_O_2_. This finding shows that treatment with BAY 41-2272 acts in priming macrophages, which can subsequently enhance the responses of these cells to the presence of a pathogen. Furthermore, the fact that BAY 41-2272 also generated a "reversible antioxidant response" increases its therapeutic potential because this response promotes the elimination and inhibition of excess free radicals, protecting the organism against tissue damage during infection (Gupta et al. 2004).

Despite the fact that BAY 41-2272 treatment did not induce the spontaneous release of H_2_O_2_, it induced the spontaneous production of NO. These results were expected because cGMP production involves an increase in the production of NO by calcium release (Proctor & Carpenter 2007). In other words, BAY 41-2272 provides signals to pre-activated macrophages (inducible NO synthase expression) and initiates NO production. In bovine aortic endothelial cells, YC-1 (the BAY 41-2272 precursor molecule) has been shown to release NO (Wohlfart et al. 1999). In vascular smooth muscle, BAY 41-2272 has also been reported to release endogenous NO (Teixeira et al. 2006). Other groups have already shown the strong synergism of BAY 41-2272 and NO, which stabilise the nitrosyl-haem complex to stimulate sGC activity and produce potent vasodilatory and antiplatelet effects (Stasch et al. 2001, Evgenov et al. 2006, Schmidt et al. 2008).

Because the BAY 41-2227 treatment significantly enhanced phagocytosis and NO production and primed cells for oxidative burst, we assessed its effects in peritoneal infection models. BAY 41-2272 significantly increased animal survival against *C. albicans* infection. However, this increase in survival was not observed with *S. aureus* infection. One possibility is that drug activity in vivo activates factors involved in the response to fungi, but not to Gram-positive bacteria. Another hypothesis is that treatment with BAY 41-2272 could increase the sensitivity of phagocytes to bacterial strategies of evasion.

Notably, the lowest dose of BAY 41-2272 (1 mg/kg) was more effective and the higher dose (3 mg/kg) was ineffective and it even caused a decrease in the animal survival rate. These data led us to consider that the presence of *S. aureus* drives the activation of resident cells, initiating an inflammatory process (Niska et al. 2012) that leads to an over-response, generating a condition similar to sepsis or septic shock. These acute inflammation states are, in many cases, more harmful to the body than the infection itself (Nguyen & Nguyen 2009), leading to the death of the organism.

Using a less aggressive infection model (bacterial or fungal inoculation into the footpad), our results showed that the animals treated with BAY 41-2272, similar to the model of peritoneal cavity infection, showed a better response to *C. albicans*, particularly when it was injected into the lesion. Because macrophages and neutrophils are considered to be the main cells involved in host defence against infections by *Candida* species (D'ostiani et al. 2000), we can again observe the action of the drug on phagocytes. Additional studies focusing on the involvement of Toll-like receptors, dectin receptor 1 and Fc receptors are required to further understand the mechanisms of macrophage activation by BAY 41-2272 in animals with *Candida* infections.

Regarding the response to *S. aureus*, we observed that the size of the paw was not changed by the BAY 41-2272 treatment regardless of whether the treatment was intralesional (SC) or systemic (IP). Because our study provided strong evidence that BAY 41-2272 activates phagocytes, we again suggest that this compound might potentiate inflammation at the site of infection. Kinsman et al. (1981) have assessed the inflammatory reactions of protein A, which is a constituent of the *S. aureus* cell wall and identified its ability to increase tissue oedema. This type of reaction caused by the aggregation of bacteria due to pro-inflammatory and pro-chemotactic responses facilitated by BAY 41-2272 can maintain the inflammatory cell influx to the site of response even after the removal of the pathogen.

In the in vivo models of infection, BAY 41-2272 was more effective in responding to the fungi compared to the bacteria. Thus, we investigated the in vitro and ex vivo microbicidal activities of peritoneal macrophages against the same pathogens. Our results showed that the in vitro treatment enhanced the microbicidal activities of the peritoneal macrophages against *C. albicans* and *S. aureus* and these increases were even more significant ex vivo. These results confirm the potential of BAY 41-2272 for treating fungal infections, specifically *C. albicans*. We also show that this treatment is effective in promoting *S. aureus* killing. These data support our hypothesis that the apparent non-resolution of *S. aureus* infection in vivo involves the maintenance of inflammation generated by the pathogen and potentiated by BAY 41-2272.

This increase in microbicidal activity is probably related to the oxidative burst, reactive nitrogen production and phagocytosis. However, we cannot exclude the possible involvement of other processes, such as phagosome pH acidification and lysosomal/granular enzyme release (Sokolovska et al. 2012), in addition to the participation of other cells. Importantly, the extensiveness of the ex vivo response indicates the relevance of chemical mediators and cells present in the physiological environment to the activation and modulation of phagocyte responses. It is likely that the action of BAY 41-2272 on other immune cells creates an environment with significantly more stimuli for macrophage activation. These data, considering a complex physiological system, provide new evidence in support of the notion that BAY 41-2272, or its pathway (sGC-cGMP), can be used as a treatment for some infections, especially in immunocompromised patients. It is important to emphasise that the cardiovascular effects of BAY 41-2272 (Thorsen et al. 2010, Joshi et al. 2011) did not limit its in vivo application.

We conclude that BAY 41-2272 causes a pro-inflammatory effect, activating mononuclear phagocytes (peritoneal macrophages). Moreover, treatment with BAY 41-2272 significantly increases mouse responses to *C. albicans* (in vivo and in vitro) and *S. aureus* (in vitro), improving peritoneal macrophage microbicidal activities against these pathogens. Our group is actively investigating the pharmacological aspects of BAY 41-2272, aiming to clarify its signalling pathways and elucidate its effects on mononuclear phagocytes. With this information, we intend to develop novel treatments to increase the quality of life of patients susceptible to infections, especially those with PID.
